# Effectiveness of a Participatory Program on Pesticide Use Behavior and Blood Cholinesterase Levels in Chiang Mai Province, Northern Thailand

**DOI:** 10.1155/2021/6746367

**Published:** 2021-11-23

**Authors:** Nootchakarn Sawarng, Surat Hongsibsong, Ratana Sapbamrer, Anurak Wongta, Phannika Tongjai

**Affiliations:** ^1^Master's Degree Program in Public Health, Faculty of Public Health, Chiang Mai University, Chiang Mai 50200, Thailand; ^2^Environment, Occupational Health Sciences and Non-Communicable Disease Center of Excellence, Research Institute for Health Sciences, Chiang Mai University, Chiang Mai 50200, Thailand; ^3^School of Health Sciences Research, Research Institute for Health Sciences, Chiang Mai University, Chiang Mai 50200, Thailand; ^4^Department of Community Medicine, Faculty of Medicine, Chiang Mai University, Chiang Mai 50200, Thailand

## Abstract

This quasiexperimental study was designed to determine the effectiveness of a participatory program on pesticide use behavior and blood cholinesterase levels. The participants were 18–60 years old, living in Thung Satok subdistrict, San Pa Tong District, Chiang Mai Province. Eighty subjects, including 32 farmers and 48 consumers, were recruited to participate in the study voluntarily by a purposive sampling technique. Data concerning each individual's behaviors were collected using questionnaires, and blood cholinesterase levels were obtained from whole blood finger, providing whole blood pre and postexperiment. The data were analyzed using Fishers' exact and paired *t*-tests, for the problem situations were independently analyzed. The results show that the participatory process made it possible to improve methods for the prevention of the unhealthy effects of pesticides. After participating in the activities, both groups showed significantly higher scores in before and after knowledge (*P* < 0.05) and a decrease in pesticide contamination in their bodies as a result of the measurement of average cholinesterase which increased significantly (*P* < 0.001). In conclusion, the participatory program was applied to solve health problems from chemical hazards. The program can raise awareness about chemical toxicity, leading to the modification of the related behavior toward chemicals and reduction of the contamination of chemicals in the body. Therefore, the adoption of participatory processes can effectively solve problems related to chemical hazards that affect health.

## 1. Introduction

A Thai population survey reported that agriculture is the main occupation of Thai people [1]. In 2020, a total of 37.90 million people were employed, including 11.61 million employed in agriculture, 9.50 million in the manufacturing sector, and 16.79 million in the service and trade sector. The data have shown that the number of agricultural workers has increased by 4.60 million since 2019 [2], which is related to the amount of pesticide imports in 2018–2020 [3]. The imported amount shows that the demand for agricultural chemicals has increased. Among them, pesticides have been widely used to reduce the problem of crop damage from pests and increase productivity [4]. Several studies have shown the misuse of pesticides and their effects to health of farmer and consumer. A report in northern Tanzania showed that most farmers usually misuse pesticides and used almost the maximum concentration of the combined pesticides and did not follow label directions or official recommendations. After using the pesticides, 68% of them fell sick with skin problems, dizziness, and headaches [5]. A study in Pakistan also reported overused and misused pesticides, especially in cotton-growing areas [6]. In addition, residual pesticides in vegetables and fruits have been reported in Thailand, which may be caused by misuse and overuse of pesticides and can lead to unhealthy effects for consumers [7]. In Thailand, using pesticides in cultivation are believed to increasing yields and reducing time in crops field. However, the residues, caused from misused pesticide of users, can affect human health and environment [8]. The effects can be found in many symptoms such as muscular, ligaments, bones and joints systems, nervous system, respiratory system, and skin [9]. The participation technique has been reported, which can be used as a solving tool in the study of using of pesticides in community [10].

Participation processes are programs that can help prevent the unhealthy impacts of pesticides. The process can be done by the community, i.e., the public recognizes its importance; jointly solves problems in accordance with the context and way of life of the community through organizing a forum to exchange knowledge; engaging in problem analysis, seeking alternatives together after collecting data; analyzing, interpreting, and summarizing data together; visualizing the problem; and solving problems together [11]. In addition, the enzyme cholinesterase is a biomarker of carbamate and organophosphate pesticide exposure. The enzyme activity decreases after exposure to carbamate and organophosphate. Therefore, it can be used as a good marker of carbamate and organophosphate exposure to determine the health effects of residual chemicals in fruits or vegetables [12].

San Pa Tong District is in Chiang Mai Province, northern Thailand. It is an important agricultural area for longan, rice, and onion cultivation where high levels of pesticides are used. Farmers and consumers living in this area are affected by pesticides, and it is necessary to reduce the pesticide exposure [13]. Herein, the present study used a participatory program for the community to understand chemical toxicity, change behaviors by using appropriate chemicals, and solve problems together. Cholinesterase activity was used as a biomarker and indicator of exposure before and after participating in the program.

## 2. Materials and Methods

### 2.1. Study Design and Subjects

The study was a quasiexperimental research study with pretest and posttest designs. The study was approved by the Human Experimentation Committee, Research Institute for Health Science, Chiang Mai University, on June 20, 2018 (document no. 37/2561). The subjects in the present study were farmers and consumers aged 18–60 years old living in San Pa Tong District, Chiang Mai Province, northern Thailand. The subjects were willing to participate in the research activity until project completion. The subjects should have had no disease that had an effect on the level of cholinesterase activity, such as liver disease, anemia, glaucoma, malnutrition, pregnancy, kidney disease, brain-related diseases, noninsulin-dependent diabetes, and muscle weakness [14].

### 2.2. Data Collection

#### 2.2.1. Questionnaire

The demographical, behavior, and pesticide exposure data were collected by a questionnaire which modified from the farmers' insecticide exposure risk assessment questionnaire by the Ministry of Public Health, Department of Disease Control, Bureau of Occupational and Environmental Diseases [14].

#### 2.2.2. Whole Blood Cholinesterase Activity Determination

Finger whole blood samples were collected in heparinized capillary tubes and transferred to the Environmental and Occupational Health Science Laboratory, Research Institute for Health Sciences, Chiang Mai University, in an ice box and stored in a −20°C freezer prior to analysis. The cholinesterase activity was measured using the method of a previous study based on the method of Ellman with some modifications. In brief, the whole blood sample was diluted in potassium phosphate buffer pH 7.4, and then, 100 *μ*l of each sample was add into the mixture of 1 : 1 acetylthiocholine iodide and 1, 5′dithiobis (2-nitrobenzoic acid) in 5.0 mM phosphate buffer (DTNB) solution in a 96-microwell plate. Measuring absorbance was performed by the kinetic method using a UV-vis spectrophotometry microplate reader, and the background absorbance was immediately measured at 405 nm, followed by 15 min of reaction. Enzyme activities were calculated by multiplying differential value of the background and 15 min absorbances with blood rection factor and reported in U/ml [15]. The first cholinesterase activity was determined before starting the participatory program as the preprogram result. The second cholinesterase activity was determined after finishing the program as a postprogram result and used to evaluate program effectiveness.

#### 2.2.3. The Participatory Program

The participatory program was modified from a model of the concept of public participation according to the concept of Cohen and Uphoff [16] to understand pesticide toxicity, change behaviors by using appropriate pesticides, and solve problems. The process of the program had 4 steps. First, the problem determination process by using the appreciation influence control (AIC) technique. The subjects participated in the workshop and used AIC techniques to identify problems, study the situation, and plan solutions for problems related to chemicals. Second, the subjects researched implementation planning steps by brainstorming ideas for the community project plans, defining roles and duties, and systematically planning activities together. Third, the follow-up steps were checked and improved, and the participants were monitored and evaluated to check the feasibility of carrying out activities; if not feasible, the work plan was amended to enable them to continue to operate according to the objectives. Fourth, the process of concluding the research entailed summarizing the study and exchanging results to improve and develop community plans. The assessment of knowledge data on pesticide use between pretest and posttest evaluations in farmers and consumers were divided into before exposure (total 9 scores), between exposure (total 24 scores), and after exposure (total 15 scores) [14].

### 2.3. Statistical Analysis

Descriptive statistics were analyzed and reported as the distribution of frequency, percentage, mean, and standard deviation. Fishers' exact tests were used to analyze behavior before and after participating in the program. A paired sample *t*-test was used for cholinesterase enzyme activity in whole blood.

## 3. Results

Demographic characteristics of subjects are given in Table 1. According to farmer and consumer groups, in the farmer group, males and females were 53.1% and 46.9%, respectively. Most of the participants were 51–60 years old, with a mean ± SD age of 54.0 ± 5.0 years, married status of 78.1%, education at the primary school level of 84.4%, and salary in the range of 0–10,000 baht of 81.3%. The consumer group results showed that the percentages of males and females were 81.2% and 18.8%, respectively. Most participants were 51–60 years old, with a mean ± SD age of 48.6 ± 9.4 years; married status, 73.0%, education at the primary school level, 52.1%, and salary in the range of 0–10,000 baht, 60.4%. Farmer had a chemical spraying behavior, 59.4%, and 40.6% worked on the use of chemicals, and all consumers did not have behavior about pesticides use.

The scores of knowledge before, between, and after expose to pesticide given in Table 2 provide that in the farmer group, the knowledge before the exposure pretest was mostly at the low level of 62.5% (score 2.36–6.08). In the posttest, most knowledge showed a moderate level of 59.4% (score 4.07–7.43). In between exposure to pesticides, it was found that for the pretest, most of them were at a moderate level of 59.4% (score 11.59–18.03), and for the posttest, most levels were 68.7% at a moderate level (score 10.68–19.26), and in knowledge after exposure to pesticides, it was found that for the pretest, it was 50.0% at a moderate level (score 5.79–12.33), and for the posttest, after participating in the activity, it was at a moderate level at 62.5% (score 5.29–11.77).

Knowledge of behavior data before use, during use, and after use/exposure to agricultural chemicals of the consumer group is as follows. The pretest data were mostly at a low level of 64.6% (score 2.51–5.61), and the posttest knowledge improved to a good level of 68.7% (score 4.53–9.93). For the knowledge between the use/exposure to agricultural chemicals, in the pretest, most of them were at a moderate level of 66.7% (score 8.37–14.97) and in the posttest at a good level of 56.2% (score 13.34–22.78). For knowledge of behavior after use/exposure to agricultural chemicals, the pretest before participating in the activity was at a moderate level of 43.8% (score 4.85–10.77) and the posttest was at a good level of 77.1% (score 8.98–15.02). According to Fishers' exact test, knowledge before, between, and after use/exposure to agricultural chemicals in the farmer group and consumer group were significantly different in knowledge before at *P* < 0.001 and after at *P*=0.008.

The results of pesticide exposure in farmers and consumers are shown as the mean ± SD of enzyme cholinesterase activity by Ellman's method to compare preprogram and postprogram values. The results showed a significant difference in the farmer group at preprogram activity of 2.17 ± 0.50 U/ml and postprogram was 2.99 ± 0.62 U/ml. Similarly, the consumer group result, which showed a significant difference at the preprogram stage, was 2.37 ± 0.57 U/ml and postprogram at 2.98 ± 0.50 U/ml as given in Table 3. Last, all results of farmers and consumers showed a significant difference at preprogram activity of 2.29 ± 0.55 U/ml and postprogram was 2.99 ± 0.61 U/ml.

## 4. Discussion

Identifying the root cause of chemical problems affecting health is another important part of the engagement process. Therefore, using AIC techniques and participation made the participant understand the chemical problem situation, brainstorm systematically, exchange guidelines, and set common goals. The participation process found activities that can be carried out continuously in 2 projects, i.e., (1) promote chemical-free vegetable cultivation for household consumption. These 2 projects from participants could reduce the risk of chemical exposure to the body. (2) Promote proper washing of vegetables before eating, in which the project activities can prevent the cause of problems by the effects of chemicals on health [17, 18]. After participating in the activity for 3 months, they had more awareness, knowledge, and understanding about the hazards of chemicals affecting health. The present study also confirms the result from previous reports. The average preexperiment scores on participants' behaviors before, during, and after using sprayed chemicals were significantly different from those of the preexperiment scores [19].

The knowledge of the good pesticide use among participants was the same direction as measuring levels of the cholinesterase activity in the blood. It was found that the participatory learning programs were proven to be effective, as shown by significant increases by the other previous studies reported [19, 20]. The behavior of subjects was changed through participation in a program to reduce the risk of pesticides as studied in shallot farmers who had increased their knowledge about the use of pesticides after joining the participate program [21, 22]. During the participation in the activities of both groups, the reduction of pesticide contamination in the body that will affect future health can be seen from the results of measurements of the amount of the enzyme cholinesterase. After participating in both groups, the mean cholinesterase levels were significantly increased, which was a different result compared with the Department of Environmental Quality Promotion; the activity of the enzyme cholinesterase was not significantly different [23].

## 5. Conclusions

In this study, the problem of incorrect pesticide use behavior was reduced, and exposure was effectively reduced. Through a participatory approach and effective collaboration from determined groups, the same behavior can be achieved. Participants find solutions to problems, plan, and work together in the community in a systematic way. The networks, both inside and outside the community, work to continually solve chemical hazard problems that meet the needs of the community. These are plans that can be further developed or can be used to solve other problems and build strength within the community to manage and resolve problems in a sustainable participatory environment.

## Figures and Tables

**Table 1:** Demographic characteristics of farmers and consumers.

Characteristics	Number (%)		Χ^2^ *P* value
	Farmers *N* = 32	Consumers *N* = 48	
Gender			9.800 (0.002)^*∗*^
Male	17 (53.1)	9 (18.8)	
Female	15 (46.9)	39 (81.2)	
Age^*∗∗*^			—
<30	0 (0.0)	4 (8.3)	
30–40	0 (0.0)	4 (8.3)	
41–50	7 (21.9)	12 (25.0)	
51–60	25 (78.1)	28 (58.4)	
Mean ± SD	54.0 ± 5.0	48.6 ± 9.4	
Status			58.833 (<0.001)^*∗*^
Single	4 (12.5)	4 (8.3)	
Married	25 (78.1)	35 (73.0)	
Widow	1 (3.1)	5 (10.4)	
Divorced	2 (6.3)	4 (8.3)	
Education			174.800 (<0.001)^*∗*^
No education	2 (6.3)	2 (4.2)	
Primary school	27 (84.4)	25 (52.1)	
Lower secondary school	0 (0.0)	6 (12.5)	
High school	2 (6.2)	10 (20.8)	
Diploma	1 (3.1)	1 (2.1)	
Bachelor or above	0 (0.0)	4 (8.3)	
Behavior about pesticides use			4.835 (0.031)^*∗*^
Spray	19 (59.4)	0 (0.0)	
Working with chemical	13 (40.6)	0 (0.0)	

^*∗*^ *P* < 0.05. ^*∗∗*^Significance using the independent *t*-test.

**Table 2:** Levels and scores of knowledges before, between, and after chemical use between pretest and posttest evaluations in both farmers and consumers.

Knowledge		Farmers (*n* = 32), number (%)		Consumers (*n* = 48), number (%)		*P* value
		Pretest	Posttest	Pretest	Posttest	
Before	Low (0–3)	20 (62.5)	5 (15.6)	31 (64.6)	13 (27.1)	0.001^*∗∗*^
	Moderate (4–6)	7 (21.9)	19 (59.4)	13 (27.1)	2 (4.2)	
	Good (7–9)	5 (15.6)	8 (25.0)	4 (8.3)	33 (68.7)	
Score (mean ± SD)		**4.22** **±** **1.86**^*∗*^	**5.75** **±** **1.68**^*∗*^	**4.06** **±** **1.55**^*∗*^	**7.23** **±** **2.70**^*∗*^	

Between	Low (0–8)	3 (9.4)	0 (0.0)	9 (18.7)	1 (2.1)	0.741
	Moderate (9–16)	19 (59.4)	22 (68.7)	32 (66.7)	20 (41.7)	
	Good (17–24)	10 (31.2)	10 (31.3)	7 (14.6)	27 (56.2)	
Score (mean ± SD)		**14.81** **±** **3.22**^*∗*^	**14.97** **±** **4.29**^*∗*^	**11.67** **±** **3.30**^*∗*^	**18.06** **±** **4.72**^*∗*^	

After	Low (0–5)	5 (15.6)	5 (15.6)	21 (43.8)	2 (4.2)	0.008^*∗∗*^
	Moderate (6–10)	16 (50.0)	20 (62.5)	11 (22.9)	9 (18.7)	
	Good (11–15)	11 (34.4)	7 (21.9)	16 (33.3)	37 (77.1)	
Score (mean ± SD)		**9.06** **±** **3.27**^*∗*^	**8.53** **±** **3.24**^*∗*^	**7.81** **±** **2.96**^*∗*^	**12.00** **±** **3.02**^*∗*^	

^*∗*^ *P* < 0.001 using the independent *t*-test. ^*∗∗*^*P* < 0.05 using Fishers' exact test.

**Table 3:** The results of cholinesterase activity in blood samples of farmers and consumers by Ellman's method.

	Farmers (*N* = 32)		Consumers (*N* = 48)		Total (*N* = 80)	
	Mean	SD	Mean	SD	Mean	SD
Preprogram (U/ml)	2.17^a^	0.50	2.37^b^	0.57	2.29^c^	0.55
Postprogram (U/ml)	2.99^a^	0.62	2.98^b^	0.50	2.99^c^	0.61

^a,b,c^Same mean differences were significant at *P* < 0.001 by the paired *t*-test.

## Data Availability

The data used to support the findings of this study are included within the article.
